# Decreased sound tolerance associated with blast exposure

**DOI:** 10.1038/s41598-019-46626-6

**Published:** 2019-07-15

**Authors:** Sarah M. Theodoroff, Kelly M. Reavis, Susan E. Griest, Kathleen F. Carlson, Tanisha L. Hammill, James A. Henry

**Affiliations:** 1 VA RR&D National Center for Rehabilitative Auditory Research (NCRAR), Veterans Affairs Portland Health Care System, Portland, Oregon USA; 2 Department of Otolaryngology/Head & Neck Surgery, Oregon Health & Science University, Portland, Oregon USA; 3 School of Public Health, Oregon Health & Science University, Portland, Oregon USA; 4 Center to Improve Veteran Involvement in Care, Veterans Affairs Portland Health Care System, Portland, Oregon USA; 5 DoD Hearing Center of Excellence (HCE), Defense Health Agency, San Antonio, Texas USA

**Keywords:** Rehabilitation, Risk factors

## Abstract

Current research on blast and other injuries sustained by United States Service members and Veterans of the Iraq and Afghanistan Wars reveals a multitude of auditory complaints linked to exposures experienced during these conflicts. Among these complaints is decreased sound tolerance, which refers to a class of auditory-related problems including physical and/or psychological reactions to aspects of everyday sounds. Limited attention has been given to the possible relationship between blast exposure and decreased sound tolerance in Service members and Veterans, which is the purpose of this report. Baseline data were gathered and analyzed from 426 Service members (n = 181) and Veterans (n = 245) who participated in the Noise Outcomes in Servicemembers Epidemiology (NOISE) Study. Logistic regression analyses were performed to generate odds ratios (ORs) with 95% confidence intervals (CIs) for each group, adjusted for age and sex. Of those who reported blast exposure, 33% of Service members (adjusted OR = 1.4; CI = 0.7–2.8) and 48% of Veterans (adjusted OR = 1.9; CI = 1.1–3.3) reported decreased sound tolerance. Among Service members and Veterans who did not report blast exposure, 28% and 34% respectively, also reported decreased sound tolerance. Overall, blast exposure increased the likelihood of participants reporting decreased sound tolerance. The strength of this association was significant in Veterans.

## Introduction

Exposure to high-intensity blasts, such as those from improvised explosive devices, can cause physical and psychological trauma^1^, including auditory impairments that persist after the immediate, often life-threatening physical injuries have been treated^2^. Auditory complaints are highly prevalent among United States Service members who served in the Iraq and Afghanistan military conflicts^3^. These complaints include hearing problems (e.g., difficulty understanding speech in complex listening environments), tinnitus, and hyperacusis^4,5^. Whereas hearing loss and tinnitus are well documented as associated with blast exposure^6^, the possible relationship between blast and decreased sound tolerance has received relatively little attention.

In this report, we examined baseline data that have been collected as part of the Noise Outcomes in Servicemembers Epidemiology (NOISE) Study, which is an on-going longitudinal study exploring the relationship between military and non-military exposures and the etiology, prevalence, and effects of auditory-related conditions among Service members and Veterans. Data collected from the NOISE Study offer the opportunity to examine the relationship between blast exposure and decreased sound tolerance in these sample populations.

## Decreased Sound Tolerance: Overview

The experience of individuals who have decreased sound tolerance is heterogeneous. There are multiple types of decreased sound tolerance; these disorders are not mutually exclusive, may not be exhaustive, and may involve similar symptoms (e.g., emotional reactions, sound-avoidant behavior, etc.) and comorbidities. Knowledge of the pathophysiology and natural history of decreased sound tolerance disorders is limited; the degree to which these disorders are the same or different phenomena is relatively unknown^7^. For these reasons, no consensus exists regarding the terminology and definitions used to describe these conditions.

The term “hyperacusis” was introduced into the medical literature by Perlman in 1938^8^. Since then, many different terms, definitions, and subtypes have been introduced^9^. For a thorough review on the history and evolution of the terminology used to describe the experience of sound intolerance, we direct the reader to Baguley and Andersson^10^. The phrase “decreased sound tolerance” was introduced by Jastreboff and Jastreboff in 2004^11^ as an umbrella term to capture the multiple types of sound intolerance disorders. Because of the variety of meanings associated with these terms, below we provide our interpretation in the form of operational definitions for the most common decreased sound tolerance disorders.

### Hyperacusis

The distinctive feature of hyperacusis is that it appears to be driven by the perceived “loudness” of external sounds. Hyperacusis is defined as an intolerance to the loudness of sounds at low-to-moderate intensity levels that would not be considered loud by most people^12^. Depending on its severity, hyperacusis can cause stress, pain, and anxiety^13^. Some individuals alter their lifestyles and social habits to avoid being around everyday sounds because exposure to these sounds can exacerbate their symptoms (e.g., pain or discomfort) and worsen their hyperacusis for an indefinite period of time^14^. There is no clinical consensus on how to diagnose hyperacusis. The most commonly used method is to base the diagnosis of hyperacusis on loudness discomfort levels (LDL) that fall below some criterion threshold in addition to patient report of decreased sound tolerance. This approach is problematic because there lacks consistency in LDL criterion thresholds used by different clinics to diagnosis hyperacusis. LDL testing does not adequately evaluate the various possible facets of hyperacusis, which may be affected by the testing method used. For some patients, just hearing the test instructions can cause emotional distress^15–18^.

### Noise sensitivity

Noise sensitivity is a multifaceted phenomenon that encompasses a range of psychological attributes and personality traits which, when taken together, contribute to the degree to which an individual is reactive to noise^19,20^. Unlike hyperacusis, noise sensitivity is not directly related to the physical intensity of sound. Kliuchlo *et al*.^21^ stress that noise sensitivity and hyperacusis are not synonymous terms and that hyperacusis is a “loudness-related hypersensitivity to sounds” whereas noise sensitivity is described as “physiological and psychological internal states, which increase the degree of reactivity to noise in general”^22^. Individuals with noise sensitivity are likely to report negative emotional reactions to multiple sounds or to the overall “noise” in an environment, rather than to specific sounds.

### Misophonia

Jastreboff and Jastreboff^23^ were the first to describe misophonia, and to distinguish it from hyperacusis. Misophonia is characterized by emotional reactions (e.g., anger, anxiety, irritability, etc.) in response to specific sounds. Additionally, there can be physiological autonomic reactions (e.g., tension) that may depend on environmental factors as well^19^. These acoustic triggers commonly include human-produced sounds (e.g., mastication, sniffling, audible breathing, whistling) and repetitive sounds (e.g., clock ticking, pen clicking, foot tapping). Schröder *et al*.^24^ describe misophonia as a cluster of symptoms that share features with other psychological disorders such as post-traumatic stress disorder (PTSD), obsessive compulsive disorder, sensory processing disorders, and phonophobia, but that it should be considered an independent disorder and diagnosed when the symptoms occur in the absence of another disorder. Two distinguishing characteristics of misophonia are the presence/anticipation of a specific sound leading to an aversive reaction and recognition of an unreasonable degree of emotional response (e.g., annoyance, anger, disgust) to the misophonic sound. Clinical guidelines do not exist and there are opposing views as to the underlying nature of misophonia and how to diagnose it. For a good summary addressing this decreased sound tolerance disorder, see Cavanna and Seri^25^.

### Phonophobia

In the field of audiology, phonophobia describes a condition for which individuals are fearful that non-damaging levels of sound will exacerbate comorbid auditory conditions (hearing loss, hyperacusis, tinnitus) and often exhibit hypervigilant behavior. In neurology, the term generally refers to *migraineur phonophobia*, a different condition describing hypersensitivity and/or aversion to sound that presents with physical discomfort associated with headache^26^. Similar to misophonia and hyperacusis, the definition of phonophobia is debated both within and across disciplines.

Overall, decreased sound tolerance manifests in many ways, but clinically we lack sensitive instruments to objectively distinguish the distinct types of decreased sound tolerance. Often, “hyperacusis” is used as an umbrella term for this class of disorders, but the vernacular used within and across disciplines (e.g., audiology, psychology, neurology) is inconsistent. On a positive note, a history of limited investigative attention to these conditions is slowly being remedied.

## Prevalence

It is difficult to estimate the prevalence of decreased sound tolerance disorders, first and foremost because there is no “gold standard” to diagnose these conditions. Related challenges include the use of different terminology and definitions, and general lack of knowledge concerning the underlying pathophysiology of the various decreased sound tolerance conditions that patients may experience.

Another issue contributing to the range in prevalence estimates reported is variation among survey questions used to evaluate decreased sound tolerance by patient self-report. Taking hyperacusis as an example, Baguley^27^ reports that prevalence estimates of hyperacusis in the general population range across countries from 6% to 17% based on data from three studies^28–30^. Inconsistent definitions across studies means that published data might over- or underestimate the actual prevalence of hyperacusis.

The purpose of the present report was to investigate the prevalence of decreased sound tolerance complaints (in general) with respect to history of blast exposure. Because there are no evidence-based metrics to differentially diagnose specific types of decreased sound tolerance, Service members and Veterans answered a single question concerning their recent experience of sound as “too loud” or “uncomfortable.”

## Methods

The NOISE Study initiated data collection in 2014 and is ongoing at two sites: (1) Department of Veterans Affairs (VA) Rehabilitation Research & Development National Center for Rehabilitative Auditory Research (NCRAR) located at the VA Portland Health Care System (VAPORHCS) in Portland, Oregon; and (2) the Department of Defense (DoD) Hearing Center of Excellence (HCE) on Joint Base San Antonio, including Wilford Hall Ambulatory Surgical Center and Brooke Army Medical Center in San Antonio, Texas.

### Participants

Service members who are currently in the military and Veterans who separated from service within approximately the last 2.5 years are eligible to participate in the NOISE Study. Recruitment methods at the NCRAR include contacting individuals who have visited VAPORHCS post-deployment health clinics, mailing recruitment letters to individuals identified from a regional web-based clinical database as potential candidates, and posting study flyers in the surrounding area. At the research site in San Antonio, recruitment methods include posting study advertisements at various military institutions in the surrounding area, handing out recruitment letters, and attending outreach events attended by large numbers of Service members.

Ethical approval for the NOISE Study is provided by the Joint Institutional Review Board (IRB) for VAPORHCS and the Oregon Health & Science University, and from the DoD Medical Research and Materiel Command IRB, for each study site, respectively. All activities were performed in accordance with the relevant IRB guidelines and regulations. All participants provided informed consent and HIPAA authorization prior to performing study-related procedures.

Participants are seen in-person at baseline and every five years thereafter with annual surveys administered by phone or mail. During the baseline visit, multiple questionnaires are administered and a comprehensive audiologic assessment is performed to address the overall aims of the NOISE Study. Data in the current report were collected between February 2014 to October 2017.

### Procedures

#### Exposure

The traumatic brain injury (TBI) and Blast Exposure History survey instrument was designed specifically for the NOISE Study to measure history of potential TBI and blast exposures. This questionnaire was adapted from the Comprehensive TBI Evaluation used clinically by the VA^31^. To capture information about blast exposure, participants are asked:

“Now we are going to ask you about your blast exposures, whether or not you experienced any injuries related to blasts. When a high explosive bomb or Improvised Explosive Device (IED) goes off, there is a ‘blast wave,’ which is a wave of highly compressed gas that hits solid objects like a person’s body and may feel almost like smashing into a wall. Do you remember experiencing this type of ‘blast wave’ or ever being told that you experienced it?”

Response options are “yes,” “not sure,” or “no.” For the current analysis, blast exposure was coded dichotomously (yes/no) with all responses of “unsure” (n = 3 Service members; n = 8 Veterans) recoded as “no.”

#### Outcome

The Tinnitus and Hearing Survey (THS)^32^ was administered to screen for decreased sound tolerance using the question, “Over the last week, sounds were too loud or uncomfortable for me when they seemed normal to others around me.” Graded response options are: “No, not a problem”; “Yes, a small problem”; Yes, a moderate problem”; “Yes, a big problem”; or “Yes, a very big problem.” Any response other than “No, not a problem” was categorized as positive for decreased sound tolerance.

### Statistical analyses

Studies comparing the overall health and health behaviors of Service members, Veterans, and civilians have established that in many ways Veterans are less healthy and have poorer health behaviors^33^. Therefore, we chose to analyze and model our results separately for Service members and Veterans.

Prevalence of decreased sound tolerance was estimated first for Service members and Veterans as separate groups and then as subgroups with and without history of blast exposure. Logistic regression analyses were performed to estimate odds ratios (OR) with 95% confidence intervals (CI) while controlling for age and sex. Covariates (i.e., age and sex) were selected a priori using a causal modeling approach to inform the hypothesized relationship between blast exposure and decreased sound tolerance^34^. Our theorized causal pathway between blast exposure and decreased sound tolerance is mediated by TBI and potentially by hearing loss and PTSD. Since these variables are on the causal pathway between blast exposure and decreased sound tolerance, TBI, hearing loss, and PTSD are not included in any of the regression models^35^.

## Results

Baseline data were analyzed from 426 participants (n = 181 Service members; n = 245 Veterans). Overall, the majority of Service members [n = 127 (70%)] and Veterans [n = 214 (87%)] were male, and both sample populations had normal hearing bilaterally defined as average thresholds at 0.25, 0.5, 1, 2 kHz (low frequency pure-tone average) and 3, 4, 6, 8 kHz (high-frequency pure-tone average) of ≤20 dB Hearing Level.

Demographic information is reported in Table 1 for Service members and Veterans according to individuals who did and did not report history of blast exposure. For our respective sample populations, 30% of Service members (54 of 181) and 44% of Veterans (108 of 245) reported history of blast exposure. In each group, blast-exposed and non-blast exposed participants were comparable based on mean age. Specifically, Service members who reported blast exposure were on average 35.8 years (SD = 8.6) compared to those without blast exposure who were on average 34.5 years (SD = 8.8). Veterans who experienced blast exposure were on average 32.9 years (SD = 8.3) compared to Veterans without history of blast exposure who were on average 33.9 years (SD = 9.5).

**Table 1:** Demographic information for the sample of Service members and Veterans.

	No Blast	Blast
Service Members (n = 181)	n = 127 (70%)	n = 54 (30%)
Age	*Mean (SD; range)*	*Mean (SD; range)*
	*34.5 (8.8; 19-60)*	*35.8 (8.6; 19-60)*
Sex	*n (%)*	*n (%)*
Male	81 (64%)	46 (85%)
Female	46 (36%)	8 (15%)
Veterans (n = 245)	n = 137 (56%)	n = 108 (44%)
Age	*Mean (SD; range)*	*Mean (SD; range)*
	33.9 (9.5; 21–61)	32.9 (8.3; 21–61)
Sex	*n (%)*	*n (%)*
Male	115 (84%)	99 (92%)
Female	22 (16%)	9 (8%)

### Prevalence of decreased sound tolerance

Prevalence of decreased sound tolerance was based on a screening question on the THS^32^ that assesses self-report of at least a “small problem” tolerating the loudness of everyday sounds. It is important to note that the participant’s affirmation to any degree of a problem does not constitute diagnosis of a “loudness” tolerance problem (i.e., hyperacusis). It simply indicates that some aspect of sound is problematic to the individual, who may judge it as annoying or intolerable to any degree. Based on the THS screening question, overall, irrespective of blast exposure, 29% (53 of 181) of Service members and 40% (99 of 245) of Veterans reported some degree of decreased sound tolerance.

While the overall prevalence of decreased sound tolerance in these samples happens to approximate the percentages of Service members and Veterans reporting exposure to blast (30% and 44%, respectively), the distribution of decreased sound tolerance prevalence across those with and without history of blast exposure varied by sample population. Compared to those without blast exposure, 33% of Service members and 48% of Veterans with a history of blast exposure reported decreased sound tolerance (see Table 2).

**Table 2:** Decreased sound tolerance prevalence and logistic regression: Unadjusted and adjusted odds ratios (OR) and 95% confidence intervals (CI) for blast exposed Service members (A) and Veterans (B).

A. Service Members (n = 181)	Decreased Sound Tolerance Prevalence, n (%)	Unadjusted OR (95% CI)	Adjusted OR (95% CI)
Blast (n = 54)	18 (33%)	1.3 (0.7–2.6)	1.4 (0.7–2.8)
No Blast (n = 127)	35 (28%)	—	—
B. Veterans (n = 245)			
Blast (n = 108)	52 (48%)	1.8 (1.1–3.0)	1.9 (1.1–3.3)
No Blast (n = 137)	47 (34%)	—	—

Adjusted models controlled for age and sex.

### Regression results

Logistic regression modeling was performed to evaluate the relationship between blast exposure and decreased sound tolerance among Service members and Veterans. Table 2 presents these models first without taking into account possible confounding variables (i.e., unadjusted OR) and also after controlling for age and sex (adjusted OR). No statistically significant association between blast and decreased sound tolerance was found among Service members (adjusted OR = 1.4; CI = 0.7–2.8). However, Veterans who experienced at least one blast were 1.9 times more likely to report decreased sound tolerance compared to Veterans without history of blast exposure (adjusted OR = 1.9; CI = 1.1–3.3). The strength of this association was significant in Veterans.

## Discussion

### Health and health behaviors

Blast exposure leads to numerous health problems that in turn increase the health burden for Service members and Veterans. Assessment of blast-related problems involves multiple disciplines, including audiology. In addition to physical injuries (e.g., eardrum rupture) that can heal relatively quickly, blast exposure is associated with multiple long-standing auditory complaints such as difficulty understanding speech in complex listening environments and auditory memory problems, often in the absence of audiometric hearing loss^36^. The focus of the current report was to estimate the frequency of reporting another type of auditory complaint, that of decreased sound tolerance, and its association with blast exposure in Service members and Veterans.

Compared to individuals without blast exposure, Service members and Veterans with a history of blast exposure more frequently reported decreased sound tolerance. The number of Service members and Veterans not exposed to blasts who also reported decreased sound tolerance is also noteworthy (28% and 34%, respectively). These prevalence estimates suggest decreased sound tolerance is not uncommon and warrants further study to better understand the nature of the problem and its functional effects.

A main finding of this report is that a stronger association between blast exposure and decreased sound tolerance was seen for Veterans than for Service members. It is important to mention that other factors not possible to control for in these analyses, such as noise exposure, might be contributing to this outcome.

Taken together, these findings underscore the need to include screening questions to identify complaints of decreased sound tolerance as part of the health assessment process. There is also a need for evidence-based diagnostic tools to aid in classifying differing types of decreased sound tolerance. A better understanding of the basis for decreased sound tolerance is needed to improve the medical and audiological assessment of these problems as well as assist in making sure appropriate referrals to health care professionals are made.

### Theoretical models

Different theories exist as to the underlying mechanism(s) for various decreased sound tolerance disorders. Hyperacusis is theorized to arise due to over-adaptive compensatory changes caused by auditory injury^37–39^, but there is no objective test for hyperacusis that can be used to confirm this theory. Distinct peripheral, subcortical, and cortical changes can all result in modulating central auditory gain, which is believed to be the underlying mechanism of hyperacusis^40^. Subcortical and cortical structures play an active role in directing attention and emotional reactions to sound. For all types of decreased sound tolerance, it is possible that emotional reactions to various sounds are likely due to enhanced connections between auditory and limbic areas of the brain. Potential neural network problems are not always accounted for in proposed conceptual models of decreased sound tolerance.

Noise sensitivity is often associated with TBI^41^, but the prevalence of noise sensitivity secondary to TBI is also not well established^42,43^. Mild TBI (i.e., concussion) may be associated with an abundance of sequelae, including noise sensitivity, collectively known as post-concussive symptoms^44^. In these instances, light sensitivity often co-occurs with noise sensitivity suggesting the underlying mechanism might not be modality specific, but rather perhaps related to impaired sensory gating. Sensory gating is a pre-attentive neurological process that functions to filter or “gate” out non-pertinent stimuli. Individuals with sensory gating problems report being overwhelmed by sensory stimuli, complaints which are associated with perceptual abnormalities^45^.

Until these knowledge gaps are explained, we are hindered in developing effective interventions to treat specific types of decreased sound tolerance disorders based on their likely sources of physiological dysfunction. For example, if the underlying mechanism giving rise to hyperacusis is primarily in the auditory domain, then audiologists could address appropriate corrective action. If the underlying issue is psychological or neurological, as is thought to be the case with misophonia and noise sensitivity, then psychologists and neurologists should be involved to direct treatment and rehabilitative care. It is important to stress that in cases of misophonia and possibly noise sensitivity, the psychoacoustic characteristics of the sound are secondary and not the primary factor triggering the negative reaction(s).

Sensitive metrics capable of identifying peripheral vs. central dysfunction associated with specific types of decreased sound tolerance disorders need to be developed and would assist in understanding the pathophysiology of each type. Findings could be useful to inform our understanding of the types of decreased sound tolerance, development of diagnostic criteria, and in the selection of appropriate rehabilitative strategies based on where impairment is found. Having this information would also advance our knowledge related to the degree these types are the same or different phenomena.

### Limitations

As noted earlier, our findings are limited by multiple challenges related to the definition and diagnosis of decreased sound tolerance. Additionally, our estimates are based on Service members’ and Veterans’ self-report. If their responses were influenced by specific recent (e.g., sirens) or memorable exposure to loud sounds, it is possible these data may be over-estimating the prevalence of decreased sound tolerance. Similarly, the singular survey question, “sounds were too loud or uncomfortable for me when they seemed normal to others around me” may not equitably capture all decreased sound tolerance conditions, particularly phonophobia. Therefore, it is possible that our findings underestimate the prevalence of decreased sound tolerance in our sample populations. Additionally, there is the possibility of recall bias with respect to blast exposure – we cannot rule out that individuals with any degree of decreased sound tolerance might also be more likely to remember or report previous blast exposures. Such recall bias, if present, could artificially inflate observed associations between blast and decreased sound tolerance^46^. Future work to validate self-report of blast exposure would strengthen research endeavors that examine adverse outcomes of blast.

This report was focused on blast as a potential cause or contributor to decreased sound tolerance. Because we theorize that both TBI and PTSD are on the causal pathway between blast exposure and decreased sound tolerance outcomes, these variables were not included in our multivariable models. Future work is also needed to examine associations between these and other risk factors for decreased sound tolerance disorders using models that are restricted to specific factors and outcomes of interest.

## Conclusions

In summary, Veterans in our sample were almost twice as likely to report decreased sound tolerance compared to those who did not report history of blast exposure. This finding is suggestive of auditory injury that may otherwise go undetected^47^. Our data show, on average, normal pure-tone hearing thresholds for both sample populations of Service members and Veterans, suggesting that referral to an audiologist would not detect abnormal findings by administration of the standard audiologic test battery. To detect decreased sound tolerance, it may be necessary to go beyond standard procedures. It is important to note that if hyperacusis is suspected, it may necessitate modifying the presentation levels of test stimuli to minimize the possibility of exacerbating symptoms^14,18^. Tidball and Fagelson^14^ provide an excellent guide on this topic.

People with decreased sound tolerance perceive the sounds we encounter throughout our daily activities (e.g., going to the grocery store, being in a noisy restaurant) to be uncomfortable and/or overwhelming, even though these sounds are at low-to-moderate intensity levels. When everyday sounds are overwhelming, quality of life and ability to function can also be reduced; many individuals alter their lifestyles and social habits to avoid situations that would expose them to these sounds. Current clinical approaches do not capture the impact of decreased sound tolerance on health behaviors and emotions, and many individuals have difficulty articulating their complaints to health-care providers^48^. This is another reason it is critical to improve the medical evaluation of Service members’ and Veterans’ health and, when appropriate, to expand the case history to screen for decreased sound tolerance. During the case history and/or clinical interview, when decreased sound tolerance is identified, it is recommended to ask patients to give examples. If patients struggle to respond, Tidball and Fagelson^14^ suggest offering common examples of sounds associated with decreased sound tolerance. In their chapter on audiological assessment of decreased sound tolerance, they provide data collected by Anari *et al*.^49^ grouping examples into low-frequency sounds (drilling machine, traffic noise, dog barking); high-frequency sounds (rattling of dishes, child crying, paper rustling, applause, dentist’s drill, birdsong); broadband noise (TV, speech); and sudden sounds (hammering, door slamming).

Participants in the current study gave similar examples of sounds associated with decreased sound tolerance that fit into these categories. Additional examples for low-frequency sounds included crowd noise and restaurant noise. Because restaurant noise can include speech and dishes clanging, there is overlap with the other categories. Other examples given by participants not previously described by Anari *et al*.^49^ include sounds from a radio; classroom lectures (speech); children’s voices, female voices, high-pitch laughter, and the ding of a microwave.

Overall, our findings suggest further research is needed to investigate the possible causal relationship between blast exposure and the development of decreased sound tolerance, including any possible delay in onset and potential impact and effects on the daily lives of Service members and Veterans. Whereas the present report focused on the relationship between blast exposure and decreased sound tolerance in individuals with military experience, it should be emphasized that decreased sound tolerance disorders also occur in civilians.

As stated earlier, there is a lot of controversy regarding how best to define and characterize decreased sound tolerance disorders and this topic is under much debate. We encourage the reader to become familiar with the different opinions and opposing views on this topic and come to their own conclusions on how best to classify and describe these conditions. We further encourage experts who are working on solving these issues to collaborate and come to a consensus on terminology that could become standardized throughout the field.

Due to the paucity of research on decreased sound tolerance disorders, there is a strong need to address the many unanswered questions pertaining to clinical manifestations, associated risk factors, impact on functioning, and relationship to comorbid conditions in civilians, Service members, and Veterans.
